# Structure-dependent modulation of potato and corn starch gelatinization and retrogradation by Salecan

**DOI:** 10.1016/j.fochx.2026.104470

**Published:** 2026-09-19

**Authors:** Wanping Xiong, Lixian Hu, Cheng Ni, Juan Zhao, Yukun Huang, Xiao Yang, Pengfei Chen, Xianggui Chen

**Affiliations:** a School of Food and Bioengineering, Xihua University, Chengdu 610039, PR China; b Sichuan Synlight Biotech Ltd., 88 Keyuan South Road, Chengdu 610000, PR China; c Chongqing Key Laboratory of Speciality Food Co-Built by Sichuan and Chongqing, Chengdu 610039, PR China

**Keywords:** Salecan, Potato starch, Corn starch, Gelatinization, Retrogradation, In vitro digestion

## Abstract

Starch retrogradation is a critical technological challenge requiring effective mitigation. This study aimed to systematically compare how Salecan, a novel food-grade linear anionic glucan, differentially modulates gelatinization and retrogradation of potato starch (PS) and corn starch (CS) based on structural differences. Salecan reduced pasting viscosity, swelling power, and viscoelasticity of PS but increased these properties for CS, indicating structure-dependent gelatinization regulation driven by electrostatic repulsion and hydrogen bonding, respectively. During retrogradation, LF-NMR, XRD, and FTIR analyses revealed that Salecan significantly decreased gel hardness, retarded water migration, lowered relative crystallinity, and reduced short-range order in both starches; SEM revealed distinct microstructural changes. In vitro digestion indicated Salecan inhibited hydrolysis of gelatinized starch but promoted that of retrograded starch. These divergent effects appear associated with inherent starch structural properties (granule size, surface charge, and amylose content), offering potential insights for targeted tailoring of starch-based foods through polysaccharide modification.

## Highlights

• Salecan modulates potato and corn starch gelatinization via electrostatic repulsion and hydrogen bonding, respectively
• 1% Salecan optimally inhibits retrogradation of both starches by retarding water migration and reducing crystallinity
• Salecan reduces gelatinized starch digestibility but raises retrograded starch digestibility via structural regulation
• Starch traits (granule size, charge, amylose) determine divergent interactions with Salecan for targeted food use

## 1 Introduction

Starch is one of the most abundant, low-cost, renewable, and biodegradable natural carbohydrate polymers, widely derived from staple crops and serving as a core raw material in food, pharmaceutical, and functional material industries (Chen et al., 2024). In the human diet, it is a primary energy source. However, the quality and nutritional characteristics of starchy foods are determined not only by their botanical origin and molecular structure, but also, to a significant extent, by subsequent processing and storage conditions. Among the critical phenomena governing these changes, starch gelatinization and retrogradation are particularly influential, as they fundamentally shape the sensory properties, digestibility, and shelf life of starchy products (Liu et al., 2022).

Most natural starches exhibit poor resistance to retrogradation and limited shear stability following gelatinization, thus failing to meet the demands of food processing applications and final product quality standards (Hu et al., 2025). Consequently, chemical and biotechnological modification techniques are widely employed to produce modified starches (Masina et al., 2017; Zhong et al., 2022). However, driven by the demand for green, nutritious, and all-natural products, there is a growing interest in incorporating natural substances such as proteins, lipids, polyphenols and non-starch polysaccharides (Zhao et al., 2021), into starch during processing for the control of starch gelatinization and retrogradation, offering a “clean-label” alternative to traditional chemical modification. Nevertheless, such natural substance-incorporated systems often face limitations. For instance, the incorporation of protein into starch has been reported to disrupt the starch gel network and influence starch digestibility primarily due to the presence of isoelectric points in proteins, where net charge neutralization can induce undesirable aggregation or phase separation(Scott & Awika, 2023). The incorporation of lipids is often constrained by challenges like poor compatibility and instability, resulting from lipids᾿ inherent hydrophobic and easily oxidized nature (Gu et al., 2025; Krishnan et al., 2021). The introduction of polyphenols into starch-based products may impart an astringent flavor as their multiple phenolic hydroxyl groups readily interact with salivary proteins(Bennick, 2002; Soares et al., 2012; Zhao et al., 2020), thereby compromising product palatability. In contrast, non-starch polysaccharides can circumvent these drawbacks due to their colorless, odorless characteristics and stabilizing properties (Rong et al., 2022). Especially, Salecan is a novel, water-soluble, microbial anionic polysaccharide that received approval as a new food additive from the U.S. FDA in 2020 and the National Health Commission of China in 2021 (Liu et al., 2023). As a microbial fermentation-derived polysaccharide, Salecan confers distinct industrial advantages over plant- and seaweed-derived polysaccharides, its production is independent of seasonal, climatic, and geographical constraints. Structurally, it is a linear, unbranched glucan consisting of a repeating unit of seven β-1,3-linked and two α-1,3-linked glucose residues, with no side chains (Fig. S1) and a final product purity exceeding 90% (Xiu et al., 2010). This primary chemical structure distinguishes it from the majority of other anionic polysaccharides, such as xanthan gum, carrageenans, and pectin, as well as from glucans like yeast β-glucan and oat β-glucan, which typically feature highly branched backbones and/or heterogeneous branch distribution (Abdul et al., 2026; Bastos et al., 2022; Jabeen et al., 2025; Niu et al., 2023). The absence of side chains in Salecan is a critical structural attribute, as it reduces steric hindrance and thermodynamic incompatibility, thereby enabling its linear backbone to more effectively align and entangle with starch molecular chains (Fu et al., 2018; Hu et al., 2021). Meanwhile, as an anionic polysaccharide, Salecan also possesses uniformly distributed carboxyl groups (derived from succinyl and pyruvyl substituents), resulting in a consistent charge distribution that supports controllable electrostatic interactions. Given its linear, unbranched, anionic structure, Salecan is hypothesized to regulate starch gelatinization by competing for water and coating granule surfaces with a distinctive starch-interaction mode, and to inhibit retrogradation by sterically hindering chain re-association, disrupting hydrogen bond reformation, and retaining water within the gel network. Despite these promising mechanisms, experimental investigations into the specific application and interaction behavior of Salecan in starch-based systems remain notably limited. At the same time, it is well recognized that starch functionality is fundamentally shaped by source-dependent structural attributes, including granule morphology, amylose-to-amylopectin ratio, and surface charge (Ding et al., 2023; Wang et al., 2014; Zhiguang et al., 2025). Yet, whether Salecan's regulatory efficacy is similarly influenced by such structural diversity remains completely unexplored.

In this study, potato starch (PS) and corn starch (CS) were selected as two contrasting starch models to systematically investigate the structural dependence of Salecan modulation. PS and CS represent archetypal tuber and cereal starches, respectively, and differ markedly in several key structural attributes that are expected to govern polysaccharide–starch interactions. These include granule size, with PS exhibiting large oval granules ranging from 15 to 100 μm while CS consists of small polygonal granules of 5–20 μm; surface charge, where PS carries negatively charged monophosphate ester groups whereas CS is nearly electrically neutral; amylose content and architecture, with CS having a higher amylose content of 25–28% but a lower degree of polymerization than PS, which possesses a highly cross-linked granular structure; and crystalline pattern, with PS displaying a B-type pattern and CS an A-type pattern (Li et al., 2024; Park & Kim, 2023; Singh et al., 2003). These distinct structural properties form an ideal pairwise contrast model for systematically investigating the dominant structural determinants (charge, granule size, amylose content) that govern differential Salecan regulation. Accordingly, the gelatinization characteristics of Salecan-starch systems during pasting and rheological behavior were measured via rotational rheometry, and their swelling power was determined by a centrifugation-based weight method. Furthermore, changes in the properties and structure of Salecan-starch gels during storage were examined, including hardness tested by a texture analyzer, water distribution characterized via low-field nuclear magnetic resonance (LF-NMR), crystalline structure analyzed by X-ray diffraction (XRD), microstructure observed using scanning electron microscopy (SEM), and hydrolysis rate assessed by in vitro digestion simulation. This study aims to systematically investigate and compare Salecan's modulation of gelatinization and retrogradation in corn starch and potato starch, two commonly used food starches, with a focus on elucidating the mechanisms underlying their heterogeneous responses and translating these findings into practical guidance for starch-based food applications.

## 2 Materials and methods

### 2.1 Materials

PS and CS were purchased from Shanghai Shengyi Industry and Trade Co., Ltd. (Shanghai, China). Salecan (food-grade, Batch No. C2101241101, 902 g/kg sugar, 18.2 g/kg protein, 25.4 g/kg moisture, and 42.6 g/kg ash content) was obtained from Sichuan Synlight Biotech Ltd. (Chengdu, China). *α*-amylase (4000 U/g) purchased from Shanghai Titan Scientific Co. Ltd. (Shanghai, China). Amyloglucosidase (100,000 U/g) and GOD-POD purchased from Shanghai Yuanye Biotechnology Co., Ltd. (Shanghai, China). All other chemical reagents were of analytical grade. The polyethylene bag (water vapor permeance ≤15 g/(m^2^·24 h), oxygen permeance ≤4000 cm^3^/(m^2^·24 h·0.1 Mpa), tensile strength ≥10 Mpa) was purchased from Kelong Chemical Reagent Company (Chengdu, China).

### 2.2 The effects of Salecan on the gelatinization of PS and CS

#### 2.2.1 Pasting properties

Based on starch dry weight, Salecan was weighed at 0.5%, 1%, 1.5% and 2% to prepare colloidal solutions of different concentrations. Starch-Salecan suspension samples were prepared by dissolving 6% (w/v) of either PS or CS in the corresponding Salecan colloidal solution, followed by mixing at room temperature for 10 min to ensure complete homogenization (Banchathanakij & Suphantharika, 2009; Villanueva et al., 2018). Subsequently, 18 g of the starch-Salecan suspension was transferred into a dedicated fixture, and the gelatinization curves of the composite pastes were measured using an MCR-302 rheometer (Anton Paar GmbH, Austria) following our previous study (Liu et al., 2023). Parameters including peak viscosity, trough viscosity, final viscosity, setback value, and breakdown value were derived from the pasting curves.

The Salecan concentrations were selected based on preliminary experiments. Below 0.5%, the effects on starch gelatinization and retrogradation were marginal, whereas above 2%, the high viscosity and potential macroscopic phase separation (particularly in potato starch systems) hindered uniform sample preparation. These levels are also within the typical dosage range of food hydrocolloids used to modify starch texture without compromising palatability (Ramírez et al., 2015; Xiu et al., 2011).

#### 2.2.2 Differential scanning calorimetry

The thermal properties of the starch-Salecan suspension were determined using a differential scanning calorimeter (DSC; model DSC3, Mettler-Toledo, Switzerland). A 10 μL aliquot of the suspension was hermetically sealed in a standard aluminum DSC pan, with an empty pan employed as the reference. The samples were heated from 30 to 90 °C at a constant rate of 10 °C/min under a nitrogen flow of 60 mL/min (Fu et al., 2013). The onset temperature (*T*_*o*_), peak temperature (*T*_*p*_), end-set temperature (*T*_*e*_), and gelatinization enthalpy change (*ΔH*) were determined from the DSC curves using the instrument-integrated software.

#### 2.2.3 Rheological analysis

The starch-Salecan suspension was transferred to a magnetic stirrer-equipped water bath, heated at 95 °C for 30 min to yield fully gelatinized starch-Salecan paste. Rheological characterizations were performed using a Physica MCR 302 rheometer (Anton Paar, Austria). Freshly prepared gelatinized samples accurately weighed and loaded onto the 40 mm parallel plate of the rheometer, with the plate temperature stabilized at 25 °C (Kim & Yoo, 2006). Apparent viscosity (*ɧ*, mPa·s) and shear stress (*τ*, Pa) of the gelatinized pastes were measured over a shear rate (*γ*, s^−1^) range of 1 to 100 s^−1^. The resulting data were analyzed using the power-law equation as defined in Eq. (1).

(1) $$\tau =K\cdot \gamma^{n}$$

where *τ* represents shear stress (Pa), *K* denotes the consistency coefficient (Pa·S^n^), *γ* stands for shear rate(s^−1^), and *n* is the flow behavior index.

Dynamic viscoelastic properties of the freshly prepared gelatinized Salecan-starch pastes were further investigated via frequency sweep measurements. Tests were carried out at a fixed strain of 1%, within an angular frequency range of 1 to 10 rad/s (Kim & Yoo, 2006). Key viscoelastic parameters, including the storage modulus (*G′*, Pa), loss modulus *(G″*, Pa) and loss factor (tan *δ*) of each sample, were recorded for subsequent analysis.

#### 2.2.4 Swelling capacity

The prepared freshly gelatinized starch-Salecan pastes were subjected to centrifugation at 8000 r/min for 20 min to remove the surface moisture. The collected precipitate was transferred to a constant temperature drying oven (DHG-9075 A, Shanghai Yiheng Technical Co. Ltd., China) and dried to constant weight at 105 °C. The swelling ability of starch granules was calculated and expressed as the mass ratio of dry matter to the corresponding wet starch paste (Vamadevan & Bertoft, 2020).

### 2.3 The effects of Salecan on the retrogradation of PS and CS

#### 2.3.1 Retrogradation of gelatinized starch

12% (w/v) PS and CS were separately dispersed into the corresponding Salecan colloidal solutions, followed by thorough stirring at room temperature for 10 min to achieve uniform mixing. The mixture was then transferred to a water bath set at 95 °C, heated and continuously stirred for 30 min to ensure complete gelatinization. After heating, the gelatinized paste was cooled naturally to room temperature, followed by refrigerated storage at 4 °C for designated durations to prepare retrograded gel samples for subsequent characterization.

#### 2.3.2 Hardness analysis

The hardness characteristics of retrograded gels were determined by total texture analysis (TPA) (TA-XT PLUS, Stable Micro System, Britain). The standardized test parameters were set as follows: P/0.5 probe, pre-test speed of 1.5 mm /s, test speed of 1.0 mm /s, post-test speed of 1.0 mm /s, compression displacement of 5.0 mm, and trigger force of 5.0 g (Banchathanakij & Suphantharika, 2009; Satrapai & Suphantharika, 2007). Prior to texture testing, the retrograded gel samples were equilibrated at room temperature for 1 h to ensure consistent testing conditions.

#### 2.3.3 Low-field nuclear magnetic resonance

To characterize the water distribution and mobility within the retrograded gel during storage, the transverse relaxation time (T_2_) and related water state parameters were determined using a Low-Field Nuclear Magnetic Resonance (LF-NMR) analyzer (MesoMR23-040 *V*-1, Suzhou Niumag Analytical Instrument Corporation, China). Before measurement, all retrograded gel samples were equilibrated at room temperature for 1 h; the samples were then transferred into glass NMR tubes for subsequent analysis. All LF-NMR measurements were carried out using the Carr-Purcell-Meiboom-Gill (CPMG) pulse sequence, with optimized instrumental parameters set as follows: 90°pulse width of 10.48 μs, 180° pulse width of 20.48 μs, scan frequency of 200 kHz, scan points of 240,202, number of scans of 8, and number of echoes of 6000 (Zhang, Sun, et al., 2020). The T_2_ relaxation time, corresponding peak area, and peak area ratio were derived and calculated to quantify the distribution of different water states in the retrograded gel.

#### 2.3.4 X-ray diffraction

The starch-Salecan gels after 7 days of retrogradation were freeze-dried, ground into fine powder, and sieved through a 150 μm sieve to obtain uniform test powder. Subsequently, X-ray diffraction (XRD) analysis was performed using an X-ray diffractometer equipped with Cu Kα radiation source. The scanning range was set at 5°–50° (2θ), with a scanning speed of 4°/min. The relative crystallinity of each sample was calculated from the acquired diffraction patterns using MDI Jade 6.0 software (Materials Data Inc., USA) (Luo, Shen, et al., 2020; Piecyk & Domian, 2021).

#### 2.3.5 Fourier transform infrared spectroscopy

2 mg of freeze-dried powder samples and KBr (200 mg) were uniformly mixed with 200 mg of anhydrous KBr powder, and the mixture was rapidly ground to ensure homogeneity before being compressed into transparent tablets using a dedicated tablet press. Fourier transform infrared (FTIR) spectra were collected using a Spectrum Two spectrometer (Spectrum Two, PerkinElmer, USA). The testing parameters were set as follows: a resolution of 4 cm^−1^, 16 cumulative scans per sample, and a scanning wavenumber range of 4000 cm^−1^–400 cm^−1^ (Xiong et al., 2017).

#### 2.3.6 Differential scanning calorimetry

For the retrograded gel, the DSC testing parameters were set as follows: freeze-dried gel samples were heated from 20 °C to 200 °C at a constant heating rate of 10 °C /min, with a nitrogen gas flow rate of 60 mL/min (Ferrero, 2017). The retrogradation enthalpy (*ΔH*) of each sample was calculated based on the integrated peak area of the corresponding thermal curve.

#### 2.3.7 Scanning electron microscopy

The starch-Salecan gels after 7 days of retrogradation were pre-cooled at −80 °C for 24 h, followed by freeze-drying to fully remove bound moisture. The resulting freeze-dried gel samples were immediately subjected to brittle fracture in liquid nitrogen to generate smooth, uniform cross-sections. To ensure sufficient electrical conductivity, the fractured cross-sectional surfaces were coated with a thin gold layer via sputtering. Microstructural features of the cross-sections were subsequently observed and captured using a field emission scanning electron microscope (SEM， Inspect F50, FEI, USA).

### 2.4 In vitro simulated digestion analysis

In vitro simulated digestion experiments were conducted to evaluate the digestibility of native and Salecan-added starch samples. Freeze-dried powders of starch gelatinized with 1% (w/w) Salecan were collected at two time points: day 0 (freshly gelatinized) and day 7 (retrograded for 7 days); native starch without Salecan addition were prepared as the control groups simultaneously.

Exactly 500 mg of each freeze-dried sample was dispersed in 10 mL of 0.2 mol/L sodium acetate buffer (pH 5.2), followed by thorough mixing to obtain uniform suspensions. The prepared suspensions were equilibrated in a constant-temperature water bath at 37 °C for 20 min. Subsequently, 10 mL of pre-prepared mixed enzyme solution, consisting of α-amylase (280 U/mL) and amyloglucosidase (2500 U/mL), was added to initiate enzymatic hydrolysis (Raza et al., 2021). The digestion reaction was carried out in a shaking incubator at 37 °C with a shaking speed of 180 rpm. Digestion supernatants were collected at designated time intervals. After complete enzyme inactivation at each time point, the digested mixtures were centrifuged at 10,000 rpm for 5 min, and the supernatant was carefully collected. The glucose content in each digestion supernatant was quantitatively determined using a glucose oxidase-peroxidase (GOPOD) assay kit, to further calculate the starch digestion rate.

### 2.5 Statistical analysis

All experimental data were subjected to statistical analysis and graphical visualization using Origin software. One-way analysis of variance (ANOVA) was performed via SPSS software (Version 21.0, USA), followed by Duncan's multiple range test for pairwise comparisons between different treatment groups. All experiments were conducted in triplicate, and data were expressed as the mean value ± standard deviation (SD). Statistical significance was defined at a probability level of *P* < 0.05, with different letters indicating significant differences between sample group.

## 3 Results and discussion

### 3.1 Effects of Salecan on the gelatinization of PS and CS

#### 3.1.1 Pasting properties

Starch gelatinization properties are critical indicators of molecular stability and short-term retrogradation propensity during thermal processing (Liu et al., 2019). As visualized in Fig. 1A and E, PS exhibited significantly higher gelatinization viscosity than CS at equivalent Salecan dosages. This intrinsic difference originates from the distinct granular structures and molecular compositions of the two starches. As shown in Figs. S2 and S3, PS has larger granules and negatively charged monophosphate ester groups on its molecular chains (Hu et al., 2025); whereas CS has smaller granular particles and electrically neutral molecular chains (Guo et al., 2020). The phosphate groups in PS contribute to intramolecular charge repulsion, which stabilizes the swollen granule structure and enhances shear resistance during gelatinization, thereby elevating its intrinsic viscosity relative to CS (Villanueva et al., 2018). Furthermore, PS also has a higher amylose polymerization degree and highly cross-linked granular structure (Hu et al., 2025), which collectively enhance molecular entanglement and shear resistance during gelatinization.

**Fig. 1:**
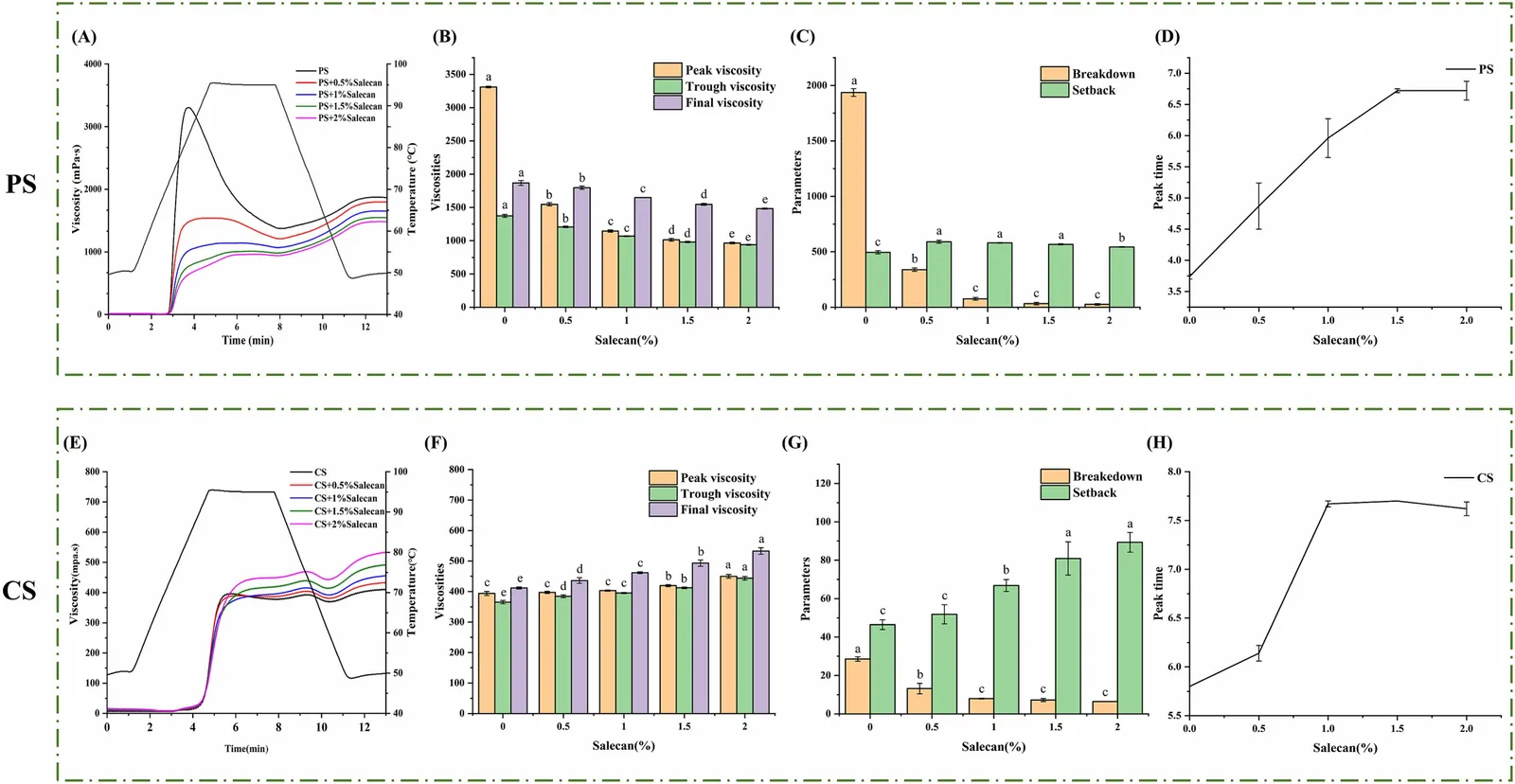
The pasting properties of starch-Salecan suspensions incorporating varying concentrations of Salecan.

Compared with native starch controls, Salecan addition shifted the gelatinization curves of both PS and CS to the right and significantly prolonged gelatinization peak time (*P* < 0.05; Fig. 1D and H), confirming its inhibitory effect on starch gelatinization. This inhibition was further verified by DSC analysis (Fig. S4 and Table S1) (Huang et al., 2021). Gelatinization can be viewed as a sequence of four events: (i) water absorption and granule hydration, (ii) granular swelling and crystalline melting, (iii) granule disruption and amylose leaching, and (iv) formation of a continuous paste. The present results indicate that Salecan acts primarily on the first three steps. Its strong water-holding capacity reduces the free water available for step (i); its surface-coating effect physically restricts water ingress and retards the crystalline melting in step (ii); and its presence in the continuous phase suppresses amylose diffusion and entanglement, thereby delaying the establishment of a viscous paste in step (iii). The contribution of electrostatic interactions to these effects depends on starch surface charge and is discussed in detail below.

Notably, Salecan exerted contrasting, dose-dependent effects on the gelatinization viscosity of PS and CS. For PS, Salecan addition significantly reduced peak, trough and final viscosity (Fig. 1B), with sharp declines observed at 2% addition. This viscosity reduction is likely to stem from electrostatic repulsion between negatively charged PS and Salecan, which may confine Salecan to the PS granule surface, inhibiting water swelling and amylopectin leaching without penetrating the granule interior (Horstmann et al., 2018; Shen et al., 2025). Conversely, Salecan significantly increased the peak viscosity, breakdown strength and final viscosity of CS (Fig. 1F), consistent with previous studies (Lin et al., 2021; Zhang, Li, et al., 2020). Unlike the electrostatic repulsion occurred in the PS-Salecan system, Salecan tends to penetrate in the aqueous phase of CS paste, likely raising colloidal concentration and bulk viscosity (Chen et al., 2016). Moreover, CS possesses a high amylose content (25%–28%) (Guo et al., 2020), which supplies abundant hydroxyl groups to form extensive hydrogen bonds with Salecan. This may reinforce molecular entanglement and network structure and further increase paste viscosity.

In addition, Salecan addition markedly reduced the breakdown values of both PS and CS pastes (Fig. 1C and G), indicating improved thermal stability of the composite systems. This finding is consistent with the delayed gelatinization peak time results observed earlier, further verifying the inhibitory effect of Salecan on starch gelatinization. Native PS and CS showed weak short-term retrogradation (setback) during cooling, but Salecan significantly increased their setback values, suggesting that Salecan regulates starch molecular rearrangement and recrystallization during cooling (Dobosz et al., 2020), which merits further long-term retrogradation research.

#### 3.1.2 Rheological properties of freshly gelatinized pastes

##### 3.1.2.1 Steady rheological properties

As shown in Fig. 2, all starch-Salecan gelatinization pastes displayed typical shear-thinning behavior, which was characterized by a decrease in apparent viscosity (*ɧ*) with an increase in shear rate (*γ*) and higher viscosity at low shear rates (Garcia-Diaz et al., 2016). This is primarily due to starch macromolecules forming aggregates through intertwining after gelatinization, hindering relative motion and flow. When an external shear force is applied to the system, the number of interlocking points between starch macromolecules decreases, reducing the flow resistance between starch molecules and leading to a significant decrease in apparent viscosity (Wang et al., 2008).

**Fig. 2:**
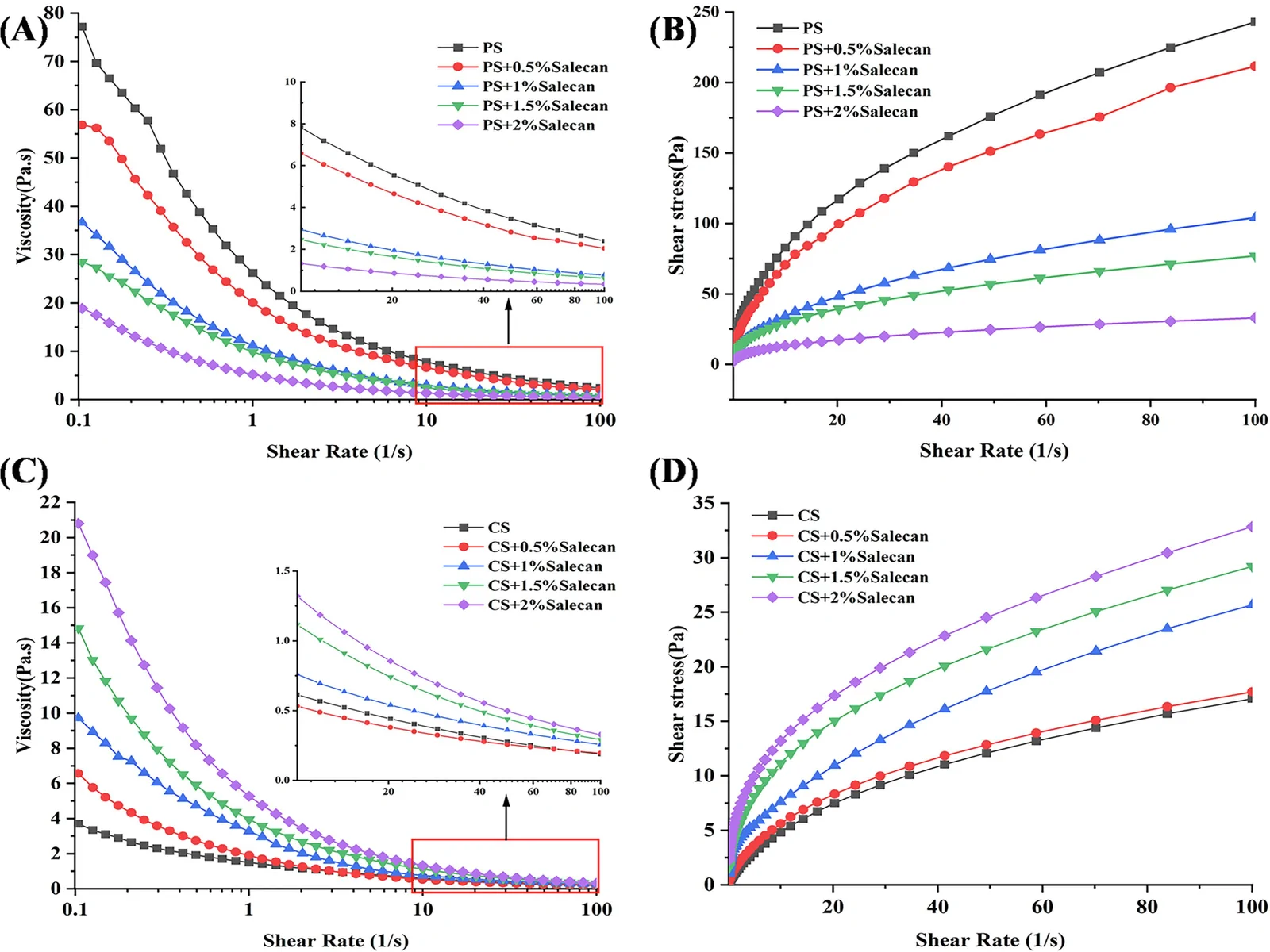
The apparent viscosity and shear stress of freshly gelatinized starch-Salecan pastes incorporating varying concentrations of Salecan: (A) apparent viscosity of PS-Salecan pastes; (B) shear stress of PS-Salecan pastes; (C) apparent viscosity of CS-Salecan pastes; (D) shear stress of CS-Salecan pastes.

Moreover, Salecan addition reduced the apparent viscosity of PS-based gelatinized pastes but increased that of CS-based ones (Fig. 2A and C). When a shear rate (*γ*) is applied to gelatinized pastes, the shear stress (*τ*) increases accordingly. However, Salecan significantly reduced the shear stress of PS (Fig. 2B) while moderately increasing that of CS (Fig. 2D). As shown in Table S2, the viscosity coefficient (K), which characterizes the viscosity of starch samples (Lin et al., 2021), was significantly reduced by Salecan in PS but increased in CS. At a 2% Salecan addition, the K value of PS decreased by 81.56%, while that of CS increased by 332.9%. This contrasting effect may be attributed to the inherent structural differences between PS and CS. Specifically, Although PS has large, porous granules with strong swelling ability, the electrostatic repulsion between Salecan and PS induces a surface barrier that inhibits its water swelling (Fig. S5) and ultimately reducing apparent viscosity. In contrast, CS exhibits a compact granular structure and high amylose content, resulting in limited water-swelling capacity. Upon incorporation of Salecan, the leached amylose interacts with Salecan through chain entanglement and hydrogen bonding, potentially promoting water retention and facilitating the formation of a reinforced composite network, thus elevating the apparent viscosity of the CS-Salecan gelatinized gels (Russ et al., 2016).

In addition, the fluid index (*n*) was <1 (R^2^ > 0.9) for all starch-Salecan systems, indicating typical shear-thinning pseudoplastic behavior (Kumar et al., 2018). Salecan enhanced pseudoplasticity in a concentration-dependent manner, enabling its use to control undesirable starch flow properties in industrial applications.

##### 3.1.2.2 Dynamic viscoelastic properties

The storage modulus (*G'*), loss modulus (*G"*), and loss tangent (tan *δ*) of starch- Salecan gelatinization samples varied with angular frequency and Salecan concentration, with the variation trend of *G"* consistent with that of viscoelasticity. Specifically, Salecan reduced the viscoelasticity of PS-based gelatinized gels (Fig. 3A) but increased that of CS-based gelatinized gels (Fig. 3C), exhibiting a significant concentration-dependent effect.

**Fig. 3:**
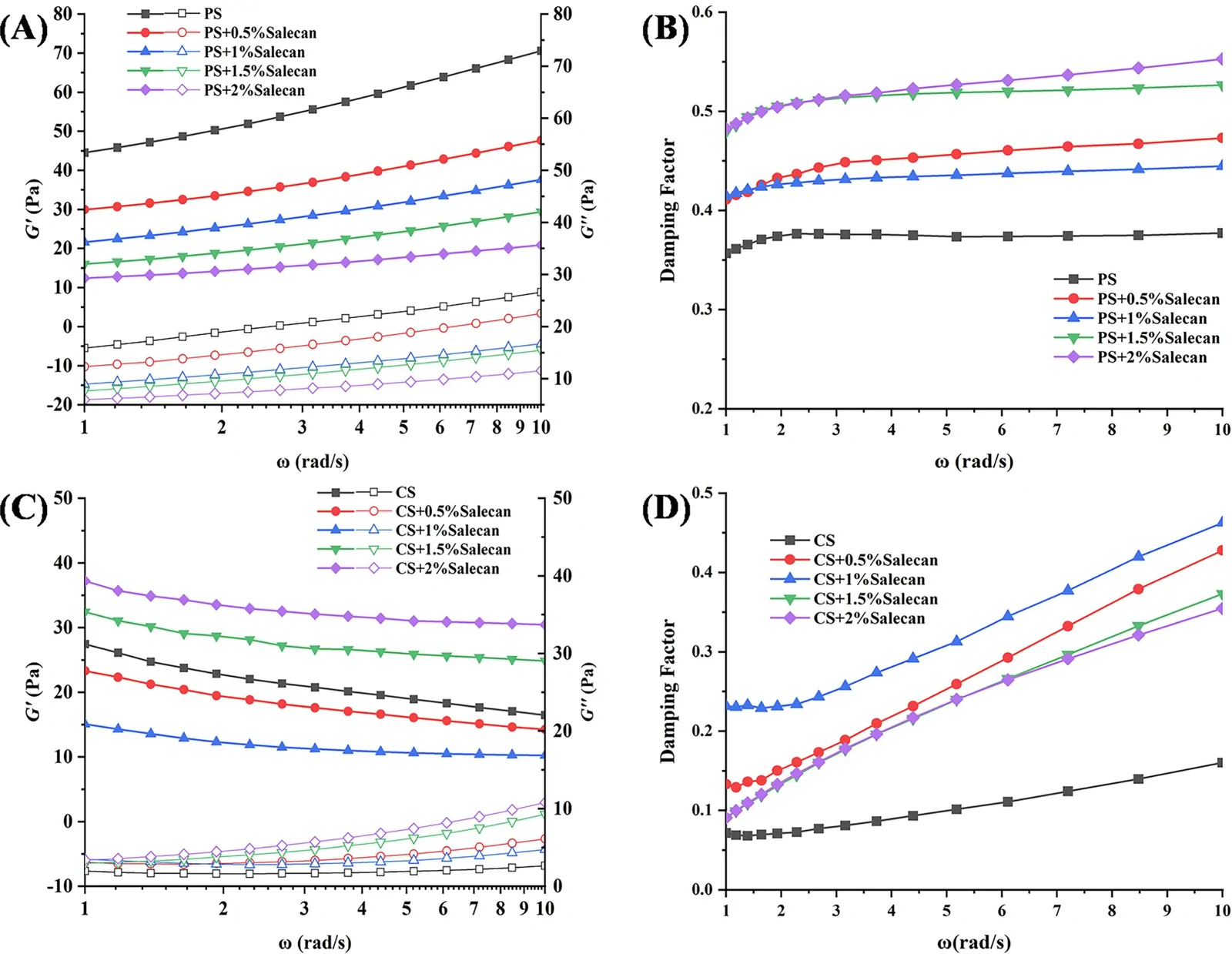
The viscoelastic properties of freshly gelatinized starch-Salecan pastes incorporating varying concentrations of Salecan: (A) *G′* and *G′′* of PS-Salecan pastes; (B) damping factor of PS-Salecan pastes; (C) *G′* and *G′′* of CS-Salecan pastes; (D) damping factor of CS-Salecan pastes (*G′* is storage modulus; *G″* is loss modulus).

For PS, Salecan addition reduced both *G'* and *G"*, which is consistent with its inhibitory effect on PS granule swelling described above, thereby reducing the amount of leached amylose available for gel network formation and lowering G' (Yin et al., 2021). For CS, *G'* first decreased and then increased with Salecan addition. It is plausible that low-dose Salecan inhibits CS gelatinization, whereas concentrations above 1% enhance molecular association and entanglement by increasing molecular density, consequently raising *G'* (Kaur et al., 2008).

Under the tested conditions, *G'* was significantly higher than *G"* and tan *δ* < 1 (Fig. 3B and D), indicating elasticity-dominated solid-like behavior (Zhu et al., 2021). Salecan increased tan *δ* in both composite gels, enhancing fluidity and reducing gel stability. This may be because Salecan forms transient, unstable junction zones that interfere with the stable starch gel network, reducing strong junctions and increasing the viscous component.

Therefore, Salecan effectively modulates PS and CS rheological properties; its addition level can be adjusted in industrial formulations to achieve desired functional properties. From a processing standpoint, the contrasting effects of Salecan on the rheological properties of PS and CS pastes provide a practical basis for tailoring starch-based formulations. For high-viscosity tuber starch pastes used in sauces, custards, and dessert gels, Salecan addition reduces apparent viscosity and shear stress, thereby improving pumpability and process fluidity without sacrificing final gel integrity. Conversely, for cereal starch systems used in soups, gravies, and bakery fillings, Salecan enhances viscosity and thermal stability, contributing to desirable mouthfeel and structural retention during hot filling and subsequent cooling. This differential modulation enables targeted selection of Salecan dosage according to starch source and desired processing performance.

### 3.2 Effects of Salecan on the retrogradation of PS and CS

#### 3.2.1 Hardness of retrograded starch gels

Gel hardness determined by TPA characterizes the resistance of a gel to deformation during initial compression, and it is a key parameter reflecting the degree of starch retrogradation (Tian et al., 2009). With prolonged storage, the hardness of all starch gels increased significantly (Fig. 4), a hallmark of starch molecular aging (Perdon et al., 2008). This aging process is primarily driven by the rearrangement of amylose chains and the recrystallization of amylopectin side chains, which synergistically drive the formation of a rigid three-dimensional network structure within the starch gel (Kaur et al., 2007).

**Fig. 4:**
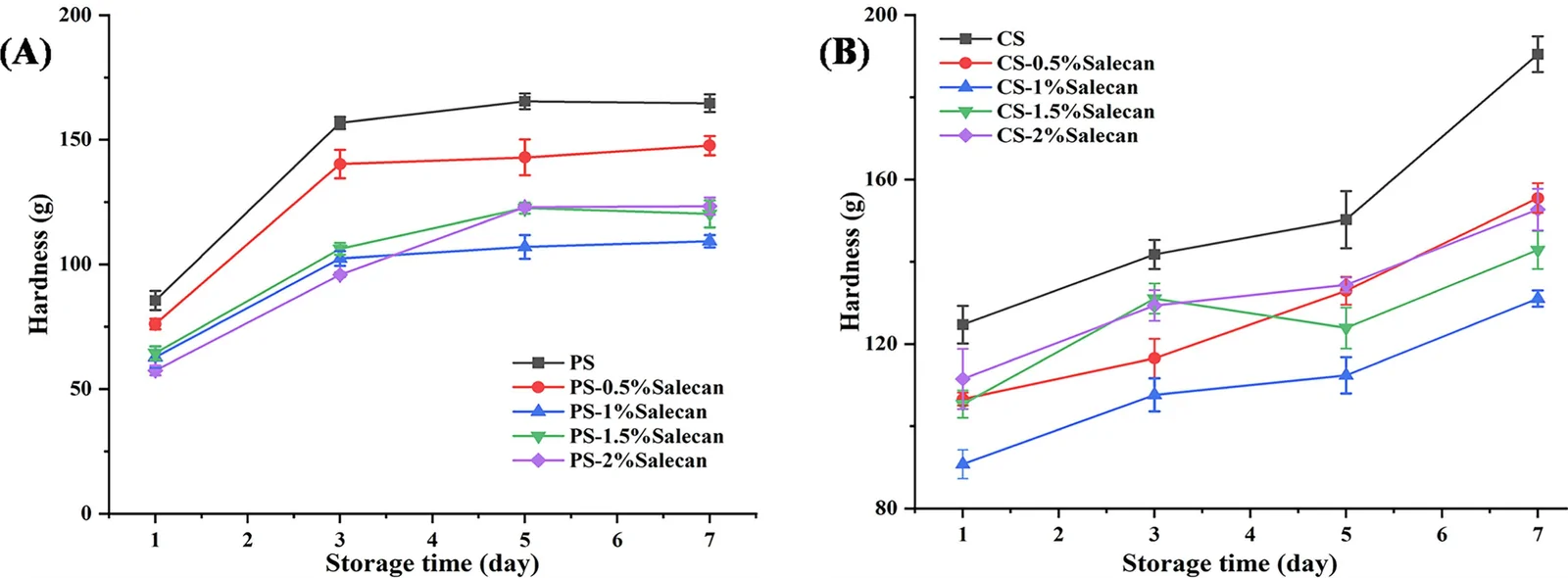
The hardness of retrograded starch-Salecan gels incorporating varying concentrations of Salecan: (A) PS-Salecan gels; (B) CS-Salecan gels.

The PS gel in the control group (Fig. 4A) exhibited lower hardness than the CS gel (Fig. 4B), a result consistent with the inherent difference in their amylose contents. A higher amylose content facilitates intermolecular hydrogen bonding and chain entanglement, thereby reinforcing the three-dimensional network structure formed during starch aging (Sandhu & Singh, 2007). The addition of Salecan significantly reduced the hardness of retrograded starch gels, with the hardness value showing a trend of first decreasing and then increasing as the Salecan dosage increased. At low concentrations, Salecan could entangle with amylose molecules, effectively inhibiting the rearrangement and aggregation of starch chains and thus leading to the formation of a loose gel network structure with reduced hardness. In contrast, at high concentrations, the strong hydrophilicity of Salecan induced competition with starch for water molecules in the system, which elevated the effective concentration of soluble amylose. This effect further promoted intermolecular association and aggregation of starch molecules, resulting in the formation of a denser gel network and ultimately causing a rebound in gel hardness (Liu et al., 2018).

At a Salecan addition level of 1%, the hardness of PS and CS gels was reduced by 33.63% and 31.19% compared with the control group, respectively, demonstrating the optimal inhibitory effect of Salecan on starch retrogradation at this dosage. This addition level is comparable to or slightly lower than the commonly reported effective concentrations for branched or sulfated anionic polysaccharides, suggesting that the strictly linear, unbranched structure of Salecan may confer a higher efficiency per unit mass in terms of chain entanglement and hydrogen bonding.

#### 3.2.2 Water distribution of retrograded starch gels

Water migration is a continuous process within starch gels during storage, and the transverse relaxation time (T_2_) measured by LF-NMR is a robust indicator for characterizing water mobility in gel systems (Marcone et al., 2013; Wei et al., 2018; Zhang, Sun, et al., 2020). A smaller T_2_ value indicates a stronger binding affinity between water molecules and the gel matrix, accompanied by lower molecular mobility (Li et al., 2016; Luo, Guo, et al., 2020).

T_2_ relaxation analysis was conducted on starch-Salecan composite gels following 21 days of storage (Fig. 5A and C) and three distinct characteristic peaks were identified in the relaxation time range of 0.01–10,000 ms, namely T_21_, T_22_, and T_23_. These peaks correspond to bound water, semi-bound water and free water in the gel matrix, respectively (Fig. 5B and D) (Ma et al., 2019), with their relative peak area ratios (A_21_, A_22_, and A_23_) reflecting the proportional distribution of the three water fractions.

**Fig. 5:**
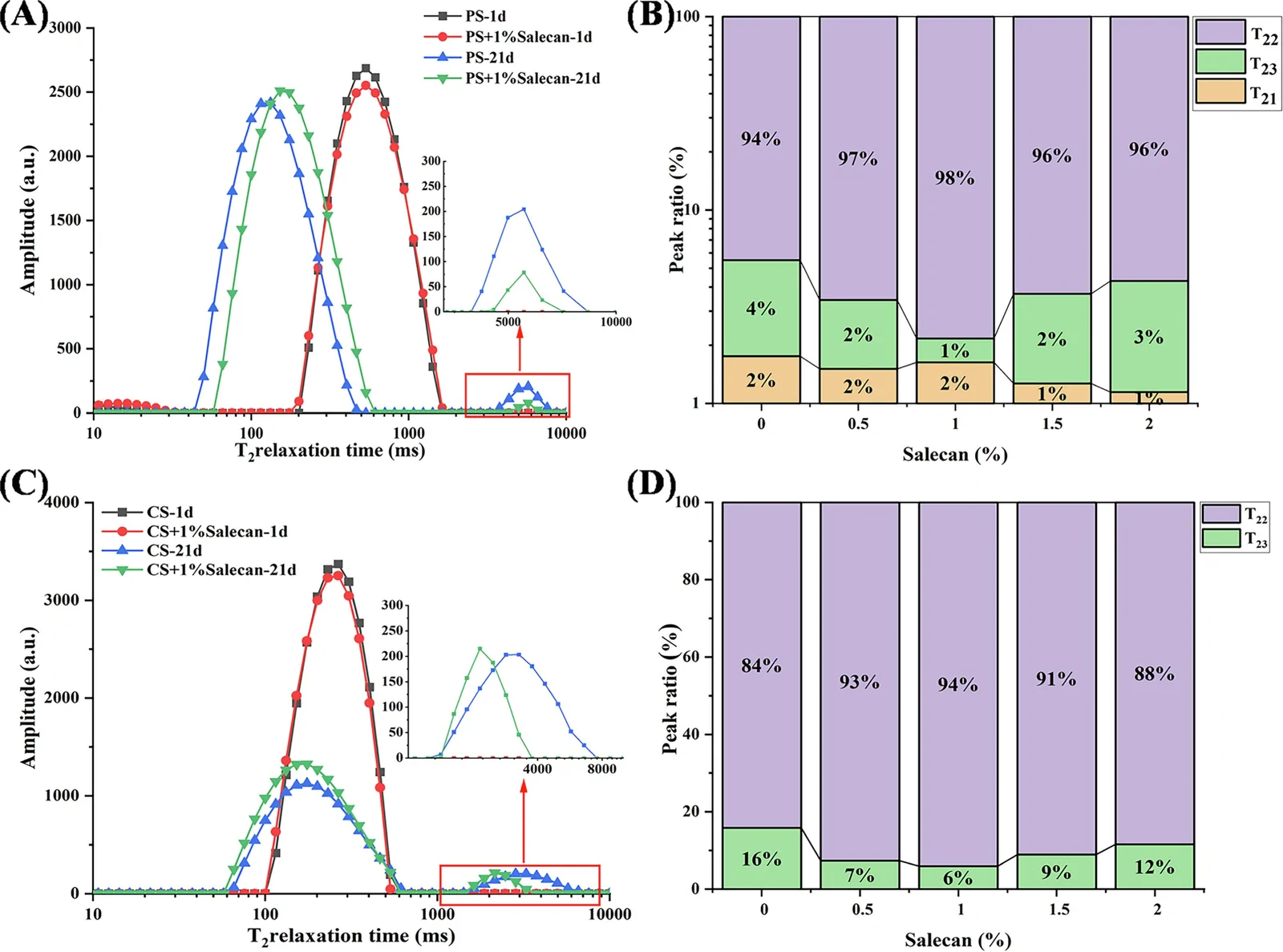
The relaxation time spectrum and water distribution of retrograded starch-Salecan gels stored 21 days: (A) Relaxation time spectrum of PS-Salecan gels; (B) Water distribution of PS-Salecan gels; (C) Relaxation time spectrum of CS-Salecan gels; (D) Water distribution of CS-Salecan gels.

With extended storage, the T_21_, T_22_, and T_23_ values of both PS and CS gels exhibited a consistent decreasing (Table S3). This phenomenon is attributed to the intermolecular cross-linking of starch chains and the molecular interactions between starch and Salecan, which jointly drive the formation of a more ordered gel network and crystalline structure. This ordered structure further restricts the migration of water molecules within the gel matrix, thus leading to the reduction in T_2_ relaxation times (Zhang et al., 2019).

Free water (T_23_) was detected in the PS gel after 14 days of storage and in the CS gel as early as 3 days of storage, with its relative proportion increasing progressively over time. This observation indicates that a portion of bound water dissociated from starch molecules during the aging process and was converted into free water, which impaired the water-holding capacity of the gels and ultimately induced water syneresis (Chen et al., 2017). This water migration process was consistent with the observed increase in gel hardness.

At a Salecan addition level of 1%, the A_23_ value of the PS-based gel decreased from 3.75% to 0.54% after 21 days of storage, while that of the CS-based gel decreased from 15.81% to 6.31% under the same conditions. The incorporation of Salecan significantly delayed the appearance of free water (T_23_) and reduced its relative content in both starch gels, indicating a potent inhibitory effect of Salecan on water migration during starch aging. This effect is presumably due to the formation of a dense three-dimensional network via cross-linking between Salecan and starch molecules, which physically entraps water molecules within the gel matrix and restricts their dissociation and migration. These results indicate that Salecan exerts a significant suppressive effect on water migration in both PS and CS gels during the aging process.

#### 3.2.3 Structure analysis of retrograded starch gels

XRD is a reliable technique for characterizing the crystallographic properties of starch, and the crystalline structures of native starch granules vary markedly with botanical source. As shown in Fig. 6A, PS presented a typical B-type crystalline structure with characteristic diffraction peaks at *2θ* = 5.5°, 17°, and 22°; whereas, CS exhibited a typical A-type crystalline structure with distinct diffraction peaks at *2θ* = 15°, 17°, 18°, 20°, and 23° (Amador-Rodríguez et al., 2020; Liu, Jiao, et al., 2021). As illustrated in Fig. 6B, the gelatinization process caused a complete disruption of the original crystalline structure of native starch granules. Moreover, XRD patterns of starch gels aged at 4 °C for 7 days with 1% Salecan incorporation revealed that the addition of Salecan did not alter the positions of the characteristic diffraction peaks of either PS or CS gels. This result indicated that Salecan had no effect on the crystalline type formed during starch retrogradation. Notably, the relative crystallinity of the starch-Salecan composite gels was significantly lower than that of the pure starch gels, suggesting that Salecan effectively inhibited the recrystallization of starch molecules during retrogradation. This observation is consistent with DSC results (Fig. S6), which showed enthalpy change associated with starch crystalline structure formation during aging was reduced in the presence of Salecan, further supporting the suppression of starch retrogradation.

**Fig. 6:**
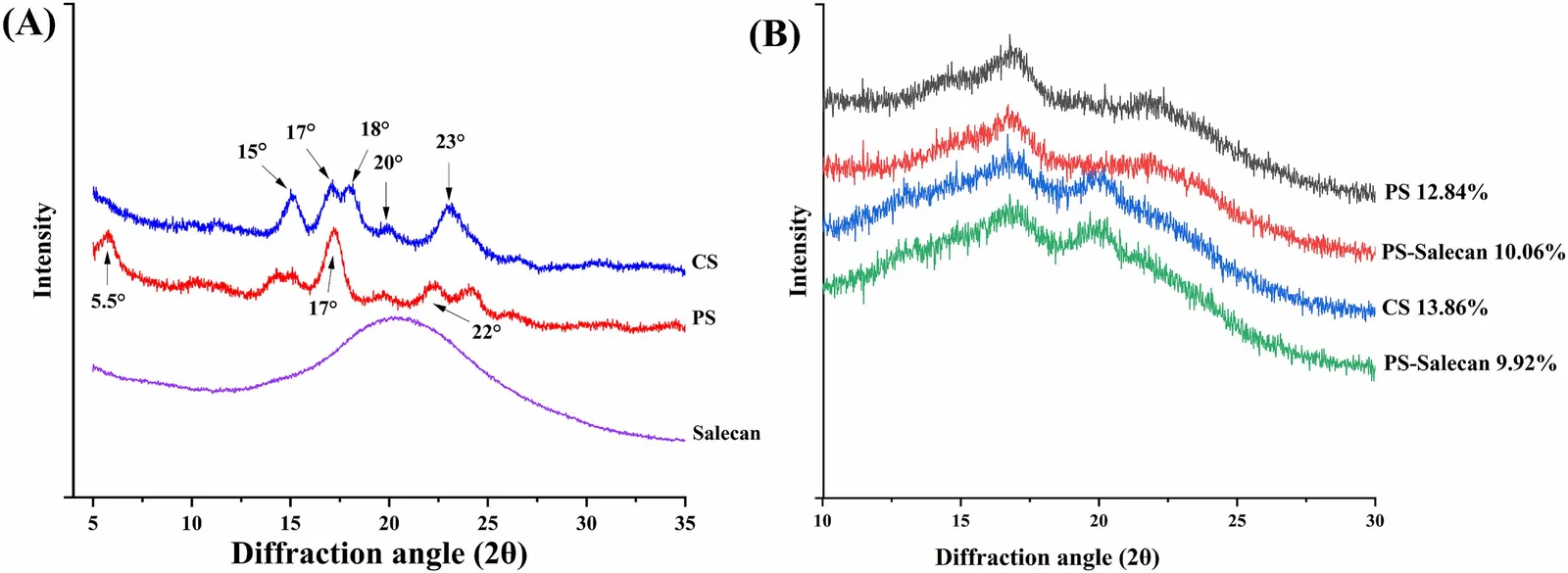
XRD analysis of native starch (A) and retrograded starch gels (B) after storage for 7 days.

In addition, FTIR spectrum of starch effectively reflects changes in chain conformation and double-helix structure (Liu et al., 2018). As shown in Fig. S7, the FTIR spectra of Salecan-starch composite gels exhibited no new characteristic absorption peaks relative to those of pure PS and CS gels, indicating the absence of covalent bond formation between Salecan and starch molecules. This observation confirms that the intermolecular interactions between the two components are predominantly mediated by hydrogen bonding (von Borries-Medrano et al., 2016).

The spectral range of 1300–800 cm^−1^ is a fingerprint region for starch, where infrared absorptions are mainly attributed to C—O and C—C stretching vibrations; these bands are highly sensitive to changes in polymer chain conformation and moisture distribution within the gel matrix (Htoon et al., 2009). Within this range, the absorption band at 995 cm^−1^ is particularly responsive to moisture content in starch systems, while the bands at 1047 cm^−1^ and 1022 cm^−1^ are associated with the short-range ordered crystalline structure and amorphous (Vandeputte et al., 2003). The ratio R_1047/1022_ is a well-recognized index for evaluating the degree of short-range molecular order in starch, and R_1022/995_ correlates closely with the formation of starch double-helical structures. Higher values of both ratios are indicative of more advanced starch aging and recrystallization. As presented in Table 1, the R_1047/1022_ and R_1022/995_ ratios of both PS and CS gels exhibited a consistent trend of first decreasing and then increasing with the elevation of Salecan addition levels. At a Salecan addition level of 1%, both ratios reached their minimum values for both starch systems, demonstrating that an optimal dosage of Salecan effectively inhibits the formation of short-range ordered structures and double-helical conformations in starch during aging.

**Table 1:** R_1047/1022_ and R_995/1022_ of Salecan-starch gels after the long-term retrogradation.

Samples	Salecan concentrations (%)	R_1047/1022_	R_995/1022_
PS	0	0.930 ± 0.002^a^	1.033 ± 0.005^a^
	0.5	0.921 ± 0.002^b^	0.910 ± 0.012^b^
	1	0.908 ± 0.001^c^	0.608 ± 0.013^c^
	1.5	0.922 ± 0.004^b^	0.896 ± 0.006^b^
	2	0.932 ± 0.004^a^	0.908 ± 0.009^b^
CS	0	0.926 ± 0.003^a^	1.055 ± 0.032^a^
	0.5	0.907 ± 0.003^c^	0.990 ± 0.018^b^
	1	0.905 ± 0.003^c^	0.603 ± 0.030^e^
	1.5	0.919 ± 0.001^b^	0.857 ± 0.005^d^
	2	0.923 ± 0.003^a^	0.939 ± 0.005^c^

Note: Different small and medium letters in the table indicate significant differences in data in the same column (*P* < 0.05).

Retrogradation proceeds through two mechanistically distinct but continuous phases: short-term amylose reassociation during cooling and long-term amylopectin recrystallization during storage, During short-term retrogradation, Salecan's linear backbone entangles with amylose and sterically hinders amylose-amylose re-association, thereby delaying initial gel network formation and reducing short-range molecular order. During long-term retrogradation, Salecan disrupts intermolecular hydrogen bond reformation required for double-helix nucleation, inhibits the ordered alignment of amylopectin side chains, and retains water within the gel matrix, thereby retarding recrystallization, reducing relative crystallinity, and mitigating gel hardening. Additionally, Salecan's strong water-holding capacity restricts water migration and impairs the plasticizing effect of water during starch aging. The inhibition of granule swelling during the gelatinization stage also indirectly reduces amylose leaching, decreasing the available molecular matrix for subsequent crystallization. Furthermore, hydrogen bonding between Salecan and the starch gel matrix reinforces a more stable composite network, further suppressing ordered recrystallization. Collectively, these synergistic effects reduce the relative crystallinity of starch gels, delay water migration, and inhibit the excessive increase in gel hardness during storage.

These anti-retrogradation effects translate into practical benefits for the long-term storage of starch-based foods. By retarding water migration and gel hardening, Salecan holds promise for improving the quality stability of chilled starch gels, frozen dough, starch-based fillings, instant noodles, and other starchy staples with extended shelf-life requirements. The reduction in syneresis, lower relative crystallinity, and maintenance of a more water-retentive network suggest that Salecan could mitigate the common textural deterioration associated with cold storage, thereby extending product acceptability.

In summary, a schematic overview of the proposed divergent gelatinization pathways and the subsequent common anti-retrogradation mechanism is presented in Fig. 7.

**Fig. 7:**
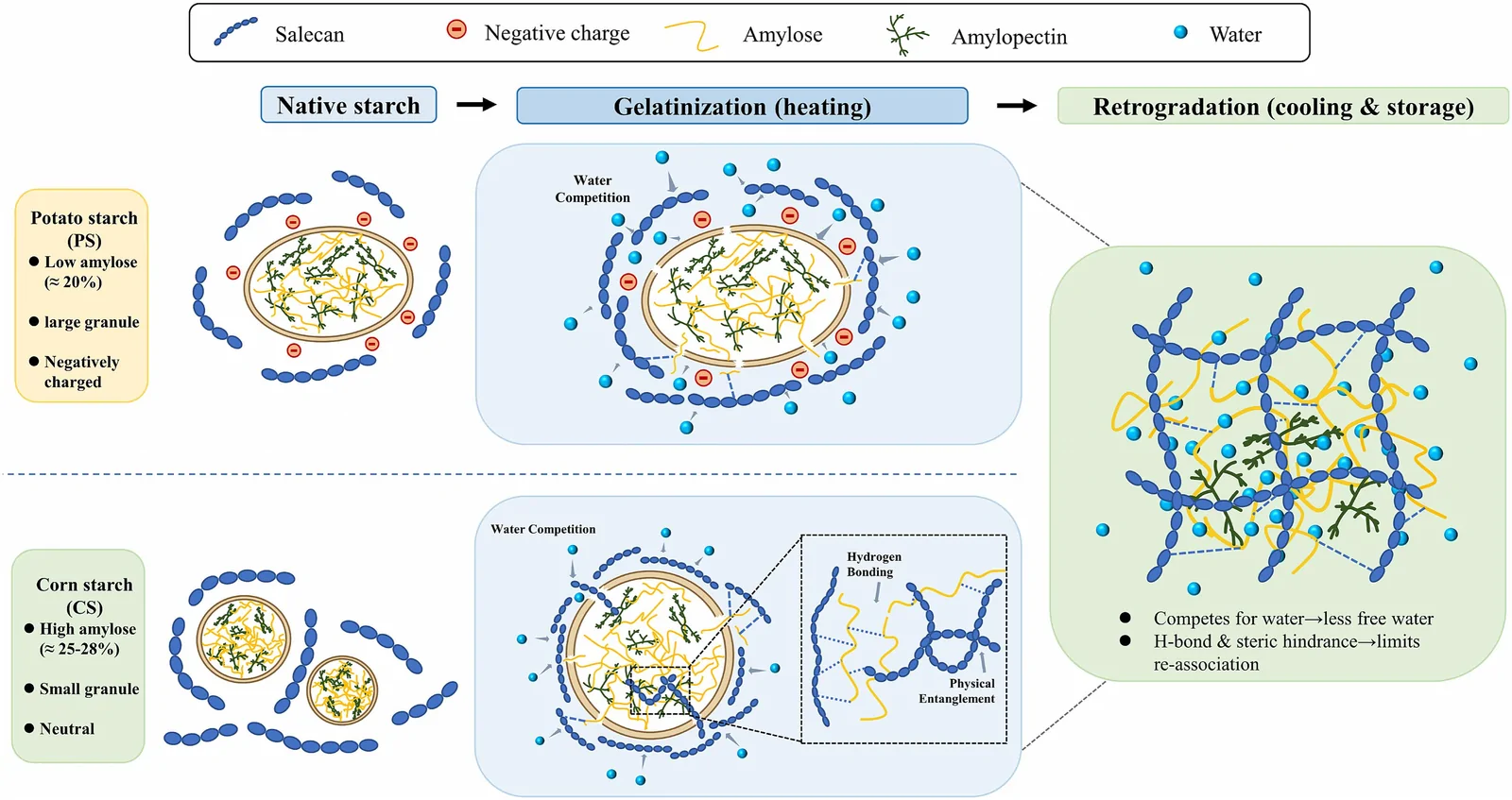
Proposed mechanism underlying the modulatory effects of Salecan on potato and corn starch properties.

#### 3.2.4 Microstructure analysis of retrograded starch gels

SEM enables direct visualization of the microstructural characteristics of starch gel cross-sections, providing intuitive morphological evidence for the inhibitory effect of Salecan on starch retrogradation. The freeze-dried pure PS and CS gel cross-sections both exhibited a disordered honeycomb-like porous structure with varying degrees of structural collapse (Fig. 8A and C). Notably, the incorporation of Salecan induced distinct microstructural modifications in PS and CS gels, with marked differences in pore size, structural order and matrix interconnection, which were closely associated with the distinct starch molecular characteristics and the differential interaction modes between Salecan and the two starch systems.

**Fig. 8:**
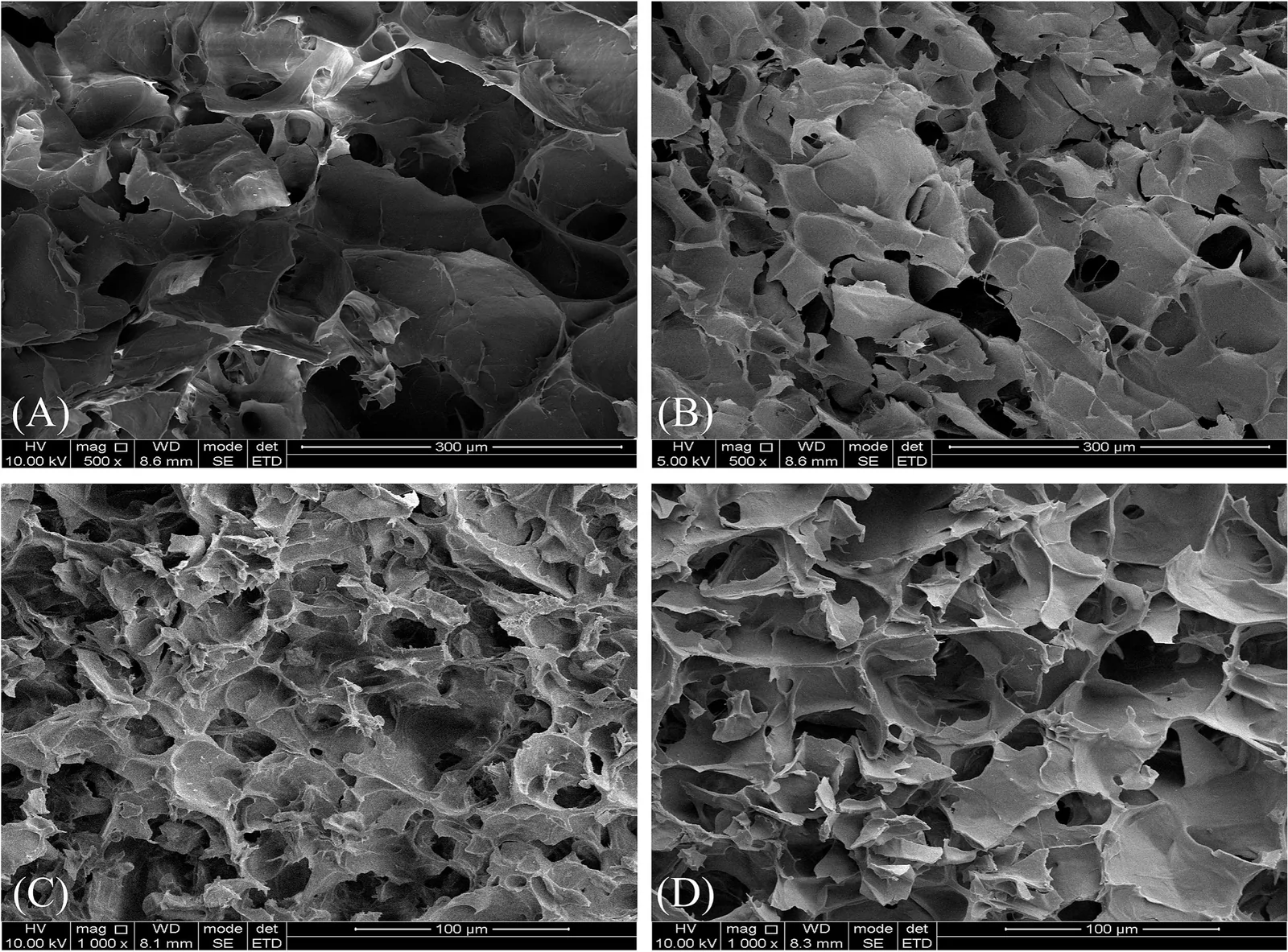
SEM images of retrograded starch gels: (A) PS gel-7d; (B) PS-Salecan gel-7d; (C) CS gel-7d; (D) CS-Salecan gel-7d.

For the PS gel, Salecan addition resulted in a significantly reduced pore size, a more ordered arrangement of the porous structure and tighter interconnections between the gel matrix networks (Fig. 8B). This morphological modification was attributed to the filling of Salecan molecules in the interstices of PS molecular chains, as well as their hydrogen-bonding interaction with leached amylose chains and low-polymerization amylopectin molecules. These interactions promoted the formation of a denser and more stable composite gel network, which effectively physically hindered the ordered rearrangement and recrystallization of PS chains during aging (Chen et al., 2017), thus inhibiting the retrogradation of the PS gel at the microstructural level. In contrast, the CS-Salecan gels exhibited a relatively smoother surface, accompanied by a significant increase in the pore size of the honeycomb-like chambers (Fig. 8D).

This morphological change was associated with the inherently higher amylose content and denser gel structure of CS, which undergoes more pronounced dehydration shrinkage during aging compared to PS. Salecan's strong water-holding capacity enhanced the water retention of the CS gel system, effectively inhibiting the dehydration shrinkage of the CS matrix during storage; while this preserved the integral microstructure of the CS gel, it led to the formation of a looser, more porous structure after freeze-drying due to the entrapment of water molecules in the composite network (Wu et al., 2010). Together, these microscopic observations support the modulation framework proposed in Fig. 7.

### 3.3 In vitro simulated digestion

The in vitro digestibility of native, gelatinized, and retrograded PS and CS gels with gradient Salecan addition levels was systematically investigated to elucidate the regulatory effect of Salecan on starch digestibility, and to further link the anti-retrogradation property of Salecan with the nutritional functional characteristics of starch-based systems.

As shown in Fig. 9A and B, a consistent digestibility trend was observed for both starch types: native starch exhibited the lowest digestibility, while gelatinized starch showed the maximum digestibility. This phenomenon is attributed to the intact, tightly packed granular structure and ordered crystalline arrangement of native starch, which form a physical barrier that hinders the accessibility of digestive enzymes to the internal starch molecules and thus inhibits enzymatic hydrolysis (Li et al., 2022). In contrast, gelatinization disrupts the native crystalline structure of starch, leading to the swelling of starch granules and leaching of amylose, which makes starch molecules more susceptible to enzymatic attack and consequently results in significantly elevated digestibility.

**Fig. 9:**
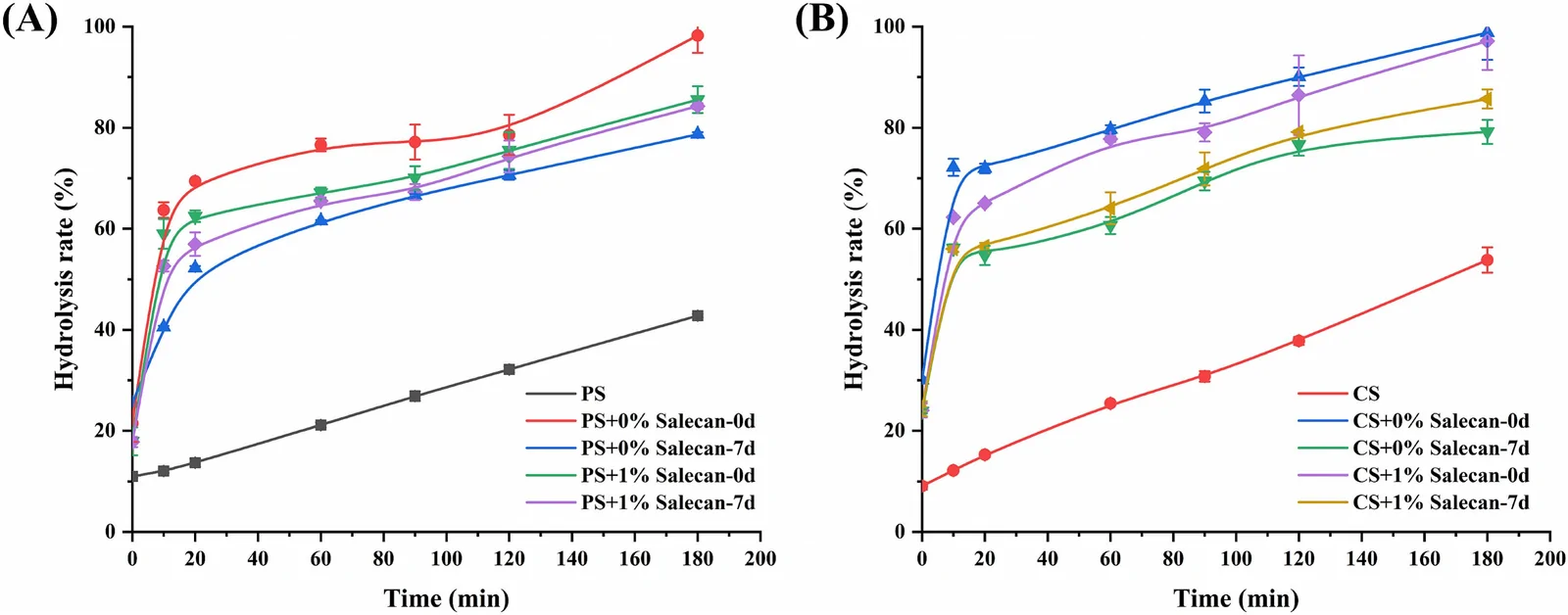
Hydrolysis rate of potato starch (A) and corn starch (B) at different Salecan addition concentrations.

The incorporation of Salecan led to a notable reduction in the digestibility of both gelatinized PS and CS gels compared with the control groups. This inhibitory effect on starch hydrolysis may be primarily ascribed to the dual action of Salecan on starch gelatinization and enzyme accessibility: on the one hand, Salecan interacts with starch molecules via hydrogen bonding and exerts a steric hindrance effect, which inhibits the complete swelling and gelatinization of starch granules during heating; on the other hand, the dense three-dimensional network formed by Salecan and starch chains physically blocks the binding sites of digestive enzymes on starch molecules, impeding the contact between enzymes and starch substrates (Li et al., 2019). More importantly, the significant reduction in the digestibility of gelatinized starch by Salecan suggests its potential application in the development of low-glycemic-index (low-GI) starch-based food products, which can slow down the rate of starch hydrolysis and glucose release in the human digestive tract, and thus provide nutritional and functional benefits for consumers, especially those with glucose metabolism disorders.

After 7 days of retrogradation at 4 °C, the digestibility of Salecan-added PS and CS gels was significantly higher than that of the pure retrograded starch gels without Salecan. This reverse trend in digestibility is closely associated with the potent anti-retrogradation effect of Salecan. Salecan disrupts the ordered recrystallization of starch molecules during aging, inhibits the formation of short-range ordered structures and double-helical conformations of starch, and reduces the relative crystallinity of retrograded starch gels. The less ordered and looser molecular structure of Salecan-modified retrograded starch gels reduces the physical resistance to digestive enzymes, thus increasing the enzymatic hydrolysis rate compared with the highly crystallized pure retrograded starch gels (Liu, Zhang, et al., 2021). This result further supports the anti-retrogradation mechanism of Salecan from the perspective of digestive physiology, suggesting that Salecan effectively suppresses the formation of resistant crystalline structures during starch aging.

From a nutritional perspective, the dual effect of Salecan—reducing the hydrolysis rate of freshly gelatinized starch while improving the digestibility of retrograded starch, which offers a novel strategy for designing starch-based foods with tailored glycemic responses. The decreased digestibility of freshly cooked starch suggests potential for developing low-glycemic-index products (e.g., steamed rice alternatives, instant porridges, or gluten-free breads), whereas the enhanced digestibility of aged starch may improve the palatability and metabolic availability of retrograded starch in stored foods. These findings position Salecan as a versatile ingredient for both health-oriented and quality-oriented starch food applications.

## 4 Conclusion

This study demonstrated that Salecan, a linear anionic glucan, exerts source-dependent regulation on starch gelatinization and retrogradation. It reduced pasting viscosity and viscoelasticity of negatively charged potato starch via electrostatic repulsion-mediated surface coating, but enhanced those of neutral high-amylose corn starch through hydrogen-bonding-facilitated molecular entanglement. At 1% (w/w, starch basis), Salecan effectively mitigated long-term retrogradation of both starches by retarding water migration, lowering relative crystallinity and short-range order, and reducing gel hardening during cold storage. In vitro digestion revealed that Salecan decreased digestibility of freshly gelatinized starches by suppressing gelatinization and limiting enzyme access, yet increased that of retrograded starches by inhibiting resistant crystal formation. These findings support the application of Salecan in chilled staples, frozen dough, ready-to-eat sauces, and starch gels to improve texture stability, reduce syneresis, extend shelf life, and guide the development of low-glycemic-index starchy foods. Given that the structural-dependence conclusions are based on two contrasting starches, future work should expand the starch panel to validate generality.

## CRediT authorship contribution statement

**Wanping Xiong:** Writing – original draft, Validation, Investigation, Data curation. **Lixian Hu:** Validation, Data curation. **Cheng Ni:** Validation, Formal analysis. **Juan Zhao:** Resources. **Yukun Huang:** Software. **Xiao Yang:** Writing – review & editing. **Pengfei Chen:** Writing – review & editing, Supervision, Methodology, Conceptualization. **Xianggui Chen:** Supervision, Project administration, Funding acquisition.

## Declaration of competing interest

The authors declare that they have no known competing financial interests or personal relationships that could have appeared to influence the work reported in this paper.

## Data Availability

Data will be made available on request.

## Appendix A Supplementary data

Article Supplements
