# Supplementary material for: Structure-dependent modulation of potato and corn starch gelatinization and retrogradation by Salecan
Source: Food Chem X. 2026 Sep 19;39:104470. doi: 10.1016/j.fochx.2026.104470 (PMC13632013; doi:10.1016/j.fochx.2026.104470)
Supplement: Supplementary file 1 [file mmc1.docx]

**Supporting Information**

**Structure-dependent modulation of potato and corn starch gelatinization and retrogradation by Salecan**

Wanping Xiong ^a^, Lixian Hu ^a^, Cheng Ni ^a^, Juan Zhao ^b^, Yukun Huang ^a, c^, Xiao Yang ^a, c^, Pengfei Chen ^a, c,^ ^*^, Xianggui Chen ^a, c,^ *

^a^ *School of Food and Bioengineering, Xihua University, Chengdu 610039, PR China*

*^b^ Sichuan Synlight Biotech Ltd., 88 Keyuan South Road, Chengdu, 610000, PR China*

*^c^ Chongqing Key Laboratory of Speciality Food Co-Built by Sichuan and Chongqing, Chengdu 610039, PR China*

* Correspondence Author.

Correspondence to: School of Food and Bioengineering, Xihua University, Chengdu, 610039, PR China.

Tel/Fax: +86-28-8772-0552

*E-mail address*: chenpengfei@xhu.edu.cn (P. F. Chen);

[chen_xianggui@mail.xhu.edu.cn](mailto:chen_xianggui@mail.xhu.edu.cn) (X. G. Chen)

**Fig. S1.** The chemical structure of Salecan.

**Fig. S2.** Particle size distribution for potato starch (PS) and corn starch (CS), respectively.

**Fig. S3.** Zeta-potential of Salecan, potato starch (PS) and corn starch (CS) in deionized water (1 mg/mL), respectively.

**Fig. S4.** DSC thermogram of starch-Salecan suspensions incorporating varying concentrations of Salecan.

**T****able S1.** Gelatinization temperature and enthalpy of starch-Salecan suspensions incorporating varying concentrations of Salecan.

| Samples | Salecan concentrations (%) | *T_o_* (℃) | *T_p_* (℃) | *T_e_* (℃) | *ΔH* (J/g) |
| --- | --- | --- | --- | --- | --- |
| PS | 0 | 61.78±0.18^c^ | 66.91±0.24^a^ | 74.00±0.45^a^ | 9.24±0.10^a^ |
|  | 0.5 | 62.01±0.07^bc^ | 66.93±0.19^a^ | 73.68±0.16^a^ | 7.44±0.26^b^ |
|  | 1 | 62.34±0.20^ab^ | 67.05±0.27^a^ | 73.87±0.31^a^ | 6.45±0.10^c^ |
|  | 1.5 | 62.24±0.09^ab^ | 66.91±0.04^a^ | 73.55±0.04^a^ | 5.99±0.07^d^ |
|  | 2 | 62.57±0.17^a^ | 67.09±0.25^a^ | 73.57±0.35^a^ | 5.77±0.10^d^ |
| CS | 0 | 63.34±0.20^c^ | 73.12±0.18^a^ | 77.35±0.55^a^ | 7.82±0.22^a^ |
|  | 0.5 | 65.89±1.47^b^ | 72.95±0.13^a^ | 77.09±0.72^a^ | 6.46±0.56^b^ |
|  | 1 | 68.40±0.33^a^ | 72.97±0.28^a^ | 78.27±1.26^a^ | 4.32±0.29^c^ |
|  | 1.5 | 65.34±0.96^b^ | 73.33±0.01^a^ | 77.18±0.71^a^ | 4.98±0.33^c^ |
|  | 2 | 66.72±0.26^ab^ | 73.36±0.06^a^ | 76.09±0.47^a^ | 4.81±0.24^c^ |

Note: Different small and medium letters in the table indicate significant differences in data in the same column (*P* < 0.05), the same as below.

As shown in **Fig. S4**, only one endothermic gelatinization peak was detected for all starch systems within 30–90℃, and the gelatinization enthalpy (*ΔH*), calculated via peak integration and reflecting the extent of starch gelatinization, was significantly reduced for both PS and CS after Salecan incorporation (**Table S1**), confirming the inhibitory effect on starch gelatinization**.**

**Table S2.** The power parameters for steady flow behavior of freshly gelatinized starch-Salecan pastes incorporating varying concentrations of Salecan.

| Samples | Salecan concentrations (%) | K (pa.s^n^) | n | R^2^ |
| --- | --- | --- | --- | --- |
| PS | 0 | 28.202±0.782^a^ | 0.470±0.005^a^ | 0.999 |
|  | 0.5 | 22.301±0.916^b^ | 0.491±0.008^a^ | 0.999 |
|  | 1 | 11.535±0.604^c^ | 0.478±0.013^a^ | 0.999 |
|  | 1.5 | 11.242±0.008^c^ | 0.417±0.004^b^ | 0.999 |
|  | 2 | 5.200±0.248^d^ | 0.400±0.008^b^ | 0.999 |
| CS | 0 | 1.225±0.044^e^ | 0.582±0.007^a^ | 0.995 |
|  | 0.5 | 1.596±0.199^d^ | 0.533±0.019^b^ | 0.996 |
|  | 1 | 2.612±0.228^c^ | 0.492±0.020^c^ | 0.996 |
|  | 1.5 | 4.102±0.133^b^ | 0.427±0.004^d^ | 0.999 |
|  | 2 | 5.303±0.045^a^ | 0.394±0.003^e^ | 0.999 |

**Fig. S5.** The swelling power of freshly gelatinized starch-Salecan pastes incorporating varying concentrations of Salecan.

**Table S3.** The water distribution of retrograded starch-Salecan gels incorporating varying concentrations of Salecan during 21 days of storage.

| Samples | Storage time (d) | T_2_ (ms) | | | A_2_ (%) | | |
| --- | --- | --- | --- | --- | --- | --- | --- |
|  |  | T_21_ | T_22_ | T_23_ | A_21_ | A_22_ | A_23_ |
| PS | 1 | 1.65±0.19^a^ | 533.67±0.00^a^ | N.D. | 2.30±0.13^a^ | 97.70±0.13^b^ | N.D. |
|  | 3 | 1.77±0.63^a^ | 433.93±30.23^b^ | N.D. | 2.21±0.10^ab^ | 97.79±0.10^b^ | N.D. |
|  | 5 | 1.43±0.35^a^ | 403.70±0.00^c^ | N.D. | 2.07±0.18^ab^ | 97.93±0.18^b^ | N.D. |
|  | 7 | 1.51±0.28^a^ | 351.12±0.00^d^ | N.D. | 1.60±0.19^bc^ | 98.40±0.19^a^ | N.D. |
|  | 14 | 0.36±0.06^b^ | 151.99±0.00^e^ | 8697.49±0.00 | 1.92±0.30^c^ | 96.99±0.24^c^ | 1.10±0.08 |
|  | 21 | 0.36±0.06^b^ | 100.00±0.00^f^ | 5722.37±0.00 | 1.75±0.04^c^ | 94.50±0.13^d^ | 3.75±0.15 |
| PS+1% Salecan | 1 | 2.49±0.08^a^ | 533.67±0.00^a^ | N.D. | 2.53±0.25^a^ | 97.47±0.25^b^ | N.D. |
|  | 3 | 2.06±0.72^a^ | 464.16±0.00^b^ | N.D. | 2.08±0.11^abc^ | 97.92±0.11^abc^ | N.D. |
|  | 5 | 2.10±0.41^a^ | 403.70±0.00^c^ | N.D. | 2.26±0.21^ab^ | 97.74±0.21^bc^ | N.D. |
|  | 7 | 1.89±0.25^a^ | 377.41±26.29^d^ | N.D. | 1.71±0.35^bc^ | 98.29±0.35^ab^ | N.D. |
|  | 14 | 0.35±0.12^b^ | 265.61±0.00^e^ | N.D. | 1.52±0.70^b^ | 98.48±0.70^a^ | N.D. |
|  | 21 | 0.42±0.07^b^ | 200.92±0.00^f^ | 6579.33±0.00 | 1.63±0.14^bc^ | 97.83±0.16^abc^ | 0.54±0.05 |
| CS | 1 | 1.70±0.34^b^ | 266.61±0.00^a^ | N.D. | 2.48±0.10^ab^ | 97.52±0.10^a^ | N.D. |
|  | 3 | 2.55±0.60^a^ | 231.01±0.00^b^ | 7564.63±534.03^a^ | 2.10±0.55^b^ | 97.86±0.54^a^ | 0.04±0.01^e^ |
|  | 5 | 0.59±0.14^c^ | 203.46±5.66^c^ | 5457.08±1423.42^b^ | 2.53±0.17^ab^ | 94.43±0.40^b^ | 3.04±0.50^d^ |
|  | 7 | 0.59±0.13^c^ | 174.75±0.00^d^ | 3764.94±0.00^c^ | 2.63±0.17^a^ | 90.96±0.45^c^ | 6.40±0.35^c^ |
|  | 14 | 0.63±0.08^c^ | 151.99±0.00^e^ | 2014.13±140.31^d^ | 2.82±0.23^a^ | 82.05±0.23^e^ | 15.130.38^b^ |
|  | 21 | N.D. | 174.75±0.00^d^ | 1873.81±0.00^d^ | N.D. | 84.19±0.19^d^ | 15.81±0.19^a^ |
| CS+1%  Salecan | 1 | 1.50±0.63^ab^ | 266.61±0.00^a^ | N.D. | 2.40±0.14^a^ | 97.60±0.14^a^ | N.D. |
|  | 3 | 2.40±1.23^a^ | 231.01±0.00^b^ | N.D. | 2.15±0.13^a^ | 97.85±0.13^a^ | N.D. |
|  | 5 | 0.81±0.18^b^ | 231.01±0.00^b^ | N.D. | 1.97±0.21^a^ | 98.03±0.21^a^ | N.D. |
|  | 7 | 0.79±0.18^b^ | 200.92±0.00^c^ | 6150.85±428.48^a^ | 2.07±0.37^a^ | 97.10±0.37^b^ | 0.83±0.18^c^ |
|  | 14 | 0.62±0.23^b^ | 161.09±11.15^d^ | 2891.03±480.66^c^ | 1.95±0.69^a^ | 93.40±0.47^d^ | 4.65±0.39^b^ |
|  | 21 | N.D. | 174.75±0.00^d^ | 3764.93±0.00^b^ | N.D. | 94.08±0.04^c^ | 5.92±0.04^a^ |

**Fig. S6.** DSC enthalpy values of retrograded starch-Salecan gels incorporating varying concentrations of Salecan after 7 days of storage.

**Fig. S7.** FTIR spectrum of retrograded starch-Salecan gels after 21 days of storage.
